# Supplementary material for: Uropathogenic Escherichia coli invade luminal prostate cells via FimH–PPAP receptor binding
Source: Nat Microbiol. 2026 Jan 8;11(2):535–50. doi: 10.1038/s41564-025-02231-0 (PMC12872464; doi:10.1038/s41564-025-02231-0)
Supplement: Supplementary file 1 — Supplementary Figs. 1–9 and source data for Supplementary Fig. 8. [file 41564_2025_2231_MOESM1_ESM.pdf]

nature microbiology

Supplementary information

https://doi.org/10.1038/s41564-025-02231-0

Uropathogenic *Escherichia coli* invade luminal prostate cells via FimH–PPAP receptor binding

In the format provided by the authors and unedited

SUPPLEMENTARY INFORMATION

Supplementary Figure 1

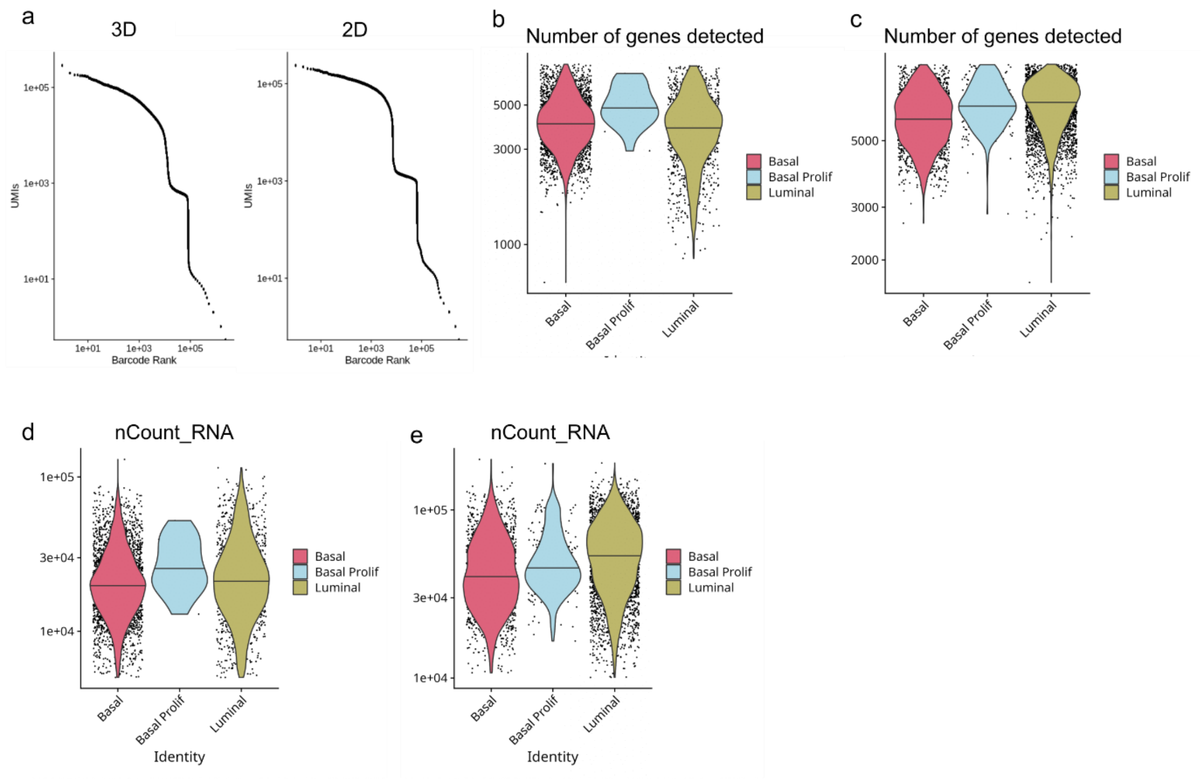

Supplementary Figure 1. Single-cell RNA-seq quality control. a. Barcode rank plots (steep curve analysis) of UMI counts of the 3D (left panel) and 2D (right panel). **b-c**. Violin plots depicting the number of gene detected for the 3D (**b**) and 2D (**c**) samples. The bar represent the median number of genes detected. **d–e.** Violin plots depicting the UMI numbers for the 3D and 2D showing total RNA content per cell for the 3D (**d**) and 2D (**e**) samples.

Supplementary Figure 2

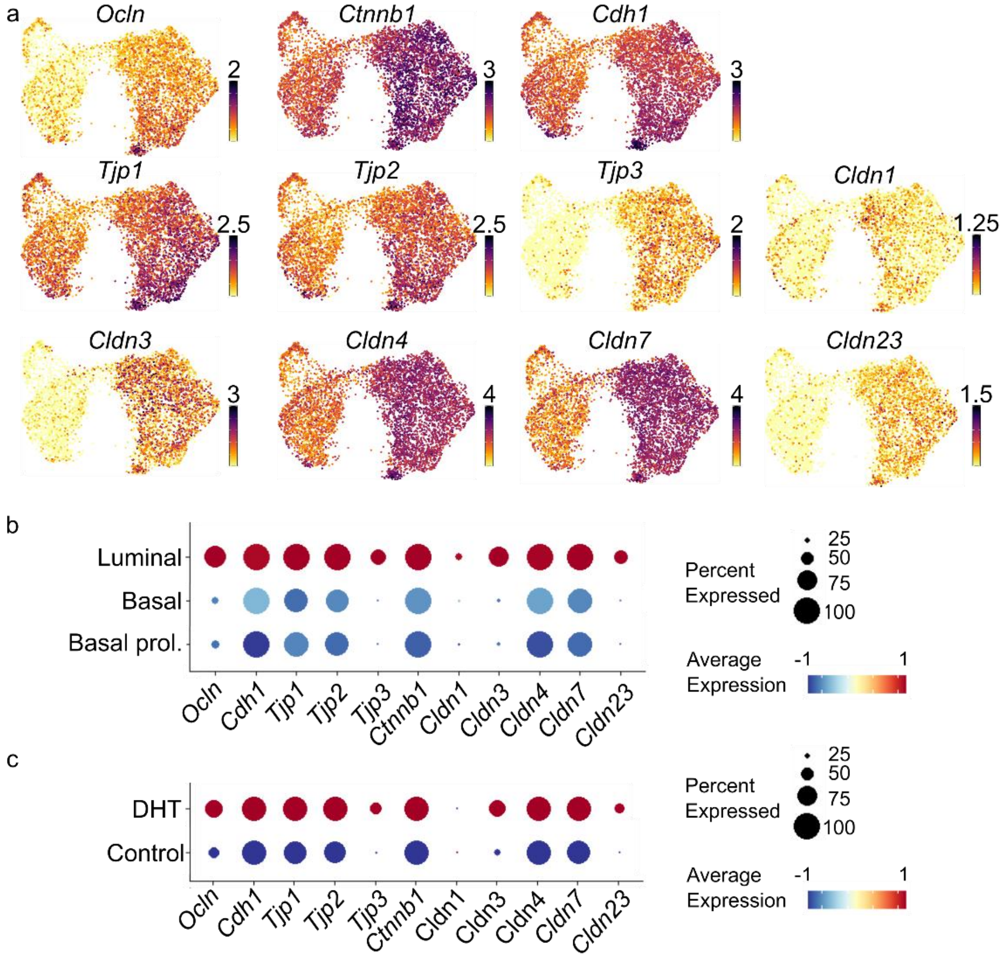

Supplementary Figure 2. The 2D organoid-based model grown in the presence of DHT (10 nM) expresses higher levels of barrier function markers. a. Expression level of markers specific for tight junction integrity colour-coded and projected on the UMAP projection of 2D organoid-derived model scRNA-seq data. **b-c.** Dot plot representation of Z-score of selected marker gene expression on 2D organoids based on cell cluster (**b**) and medium treatment (**c**, Control or 10 nM DHT).

Supplementary Figure 3

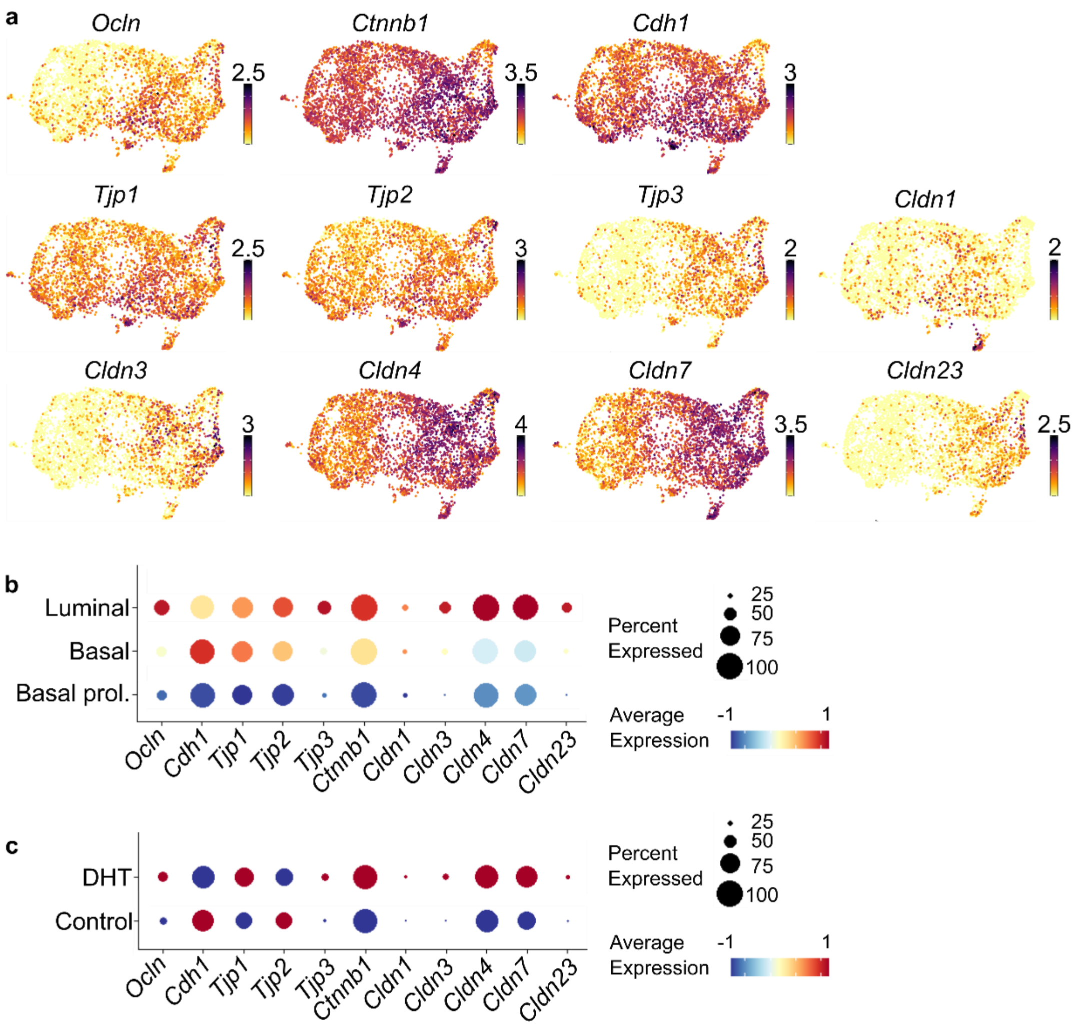

Supplementary Figure 3. 3D prostate organoids do not express high levels of barrier function markers in the presence of DHT (10 nM). a. Expression level of markers specific for tight junction integrity colour-coded and projected on the UMAP projection of 3D organoids scRNA-seq data. **b-c.** Dot plot representation of Z-score of selected marker gene expression on 3D organoids based on cell cluster (**b**) and medium treatment (**c**, Control or 10 nM DHT).

Supplementary Figure 4

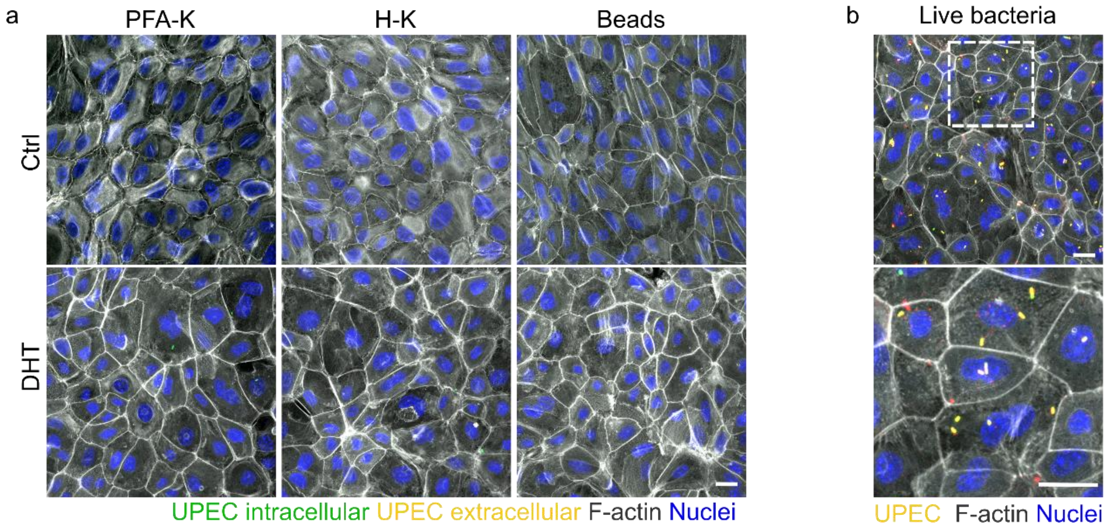

Supplementary Figure 4. PFA- or Heat-killed UPEC do not invade prostate cells. a. Representative confocal microscopy images of the organoid-based models incubated with PFA-killed (PFA-K), Heat-killed (H-K) UTI89 or fluorescent beads. F-actin was stained with phalloidin (grey) and nuclei counterstained with Hoechst 33342 (blue). Scale bar 25 µm (*n* = 3 biological replicates). **b.** Experimental control: cells grown in DHT medium were infected with live UPEC expressing GFP and stained with an anti-LPS antibody couple with a secondary Alexa 594 (red). Cells were permeabilised with Triton X-100 (0.5%) before staining to confirm selective binding of the LPS antibody. Nuclei were counterstained using Hoechst 33342 (blue) and F-actin was counterstained using phalloidin (grey). Scale bar 25 µm (*n* = 3 biological replicates).

Supplementary Figure 5

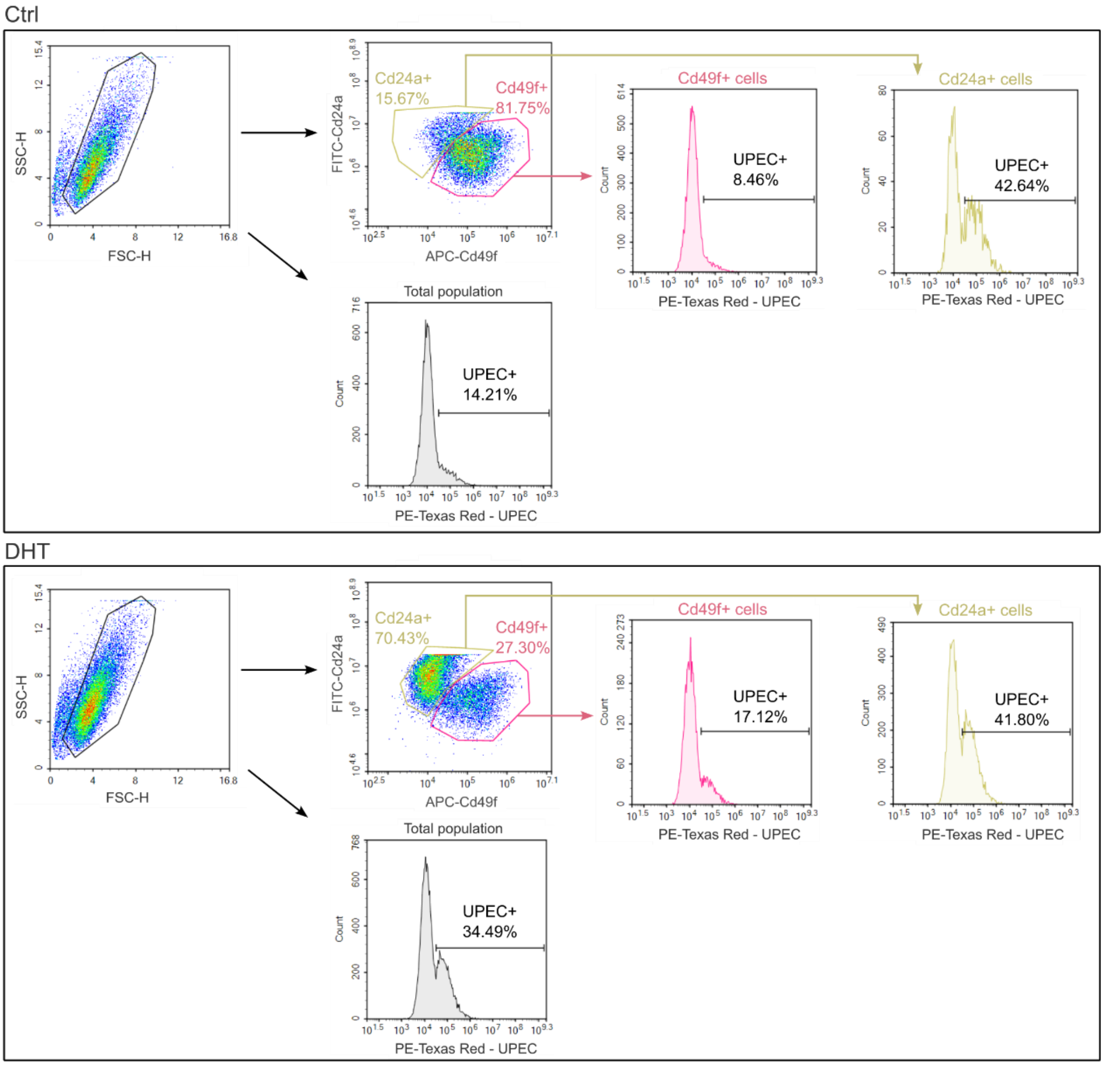

Supplementary Figure 5. Flow cytometry gating strategy. Cells were first gated on SSC versus FSC to exclude debris, then gated based on APC-CD49f (basal cells) or FITC-CD24 (luminal cells) expression. mCherry-positive cells (infected cells) were subsequently measured.

Supplementary Figure 6

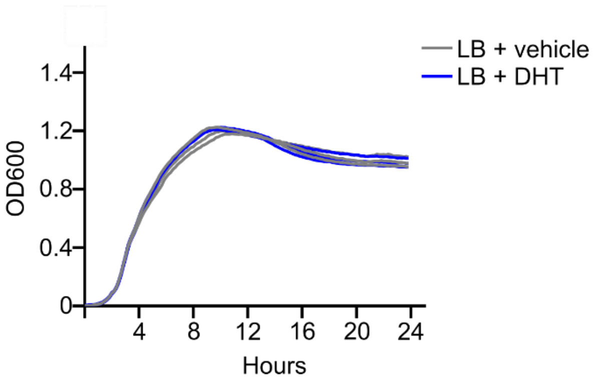

Supplementary Figure 6. DHT does not affect UPEC growth. UTI89 growth curve in LB in the presence (blue lines) or absence (grey lines) of 10 nM DHT (*n* = 3 biological replicates).

Supplementary Figure 7

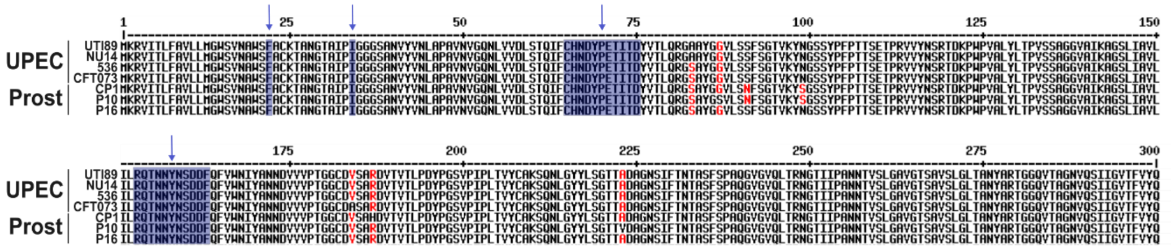

Supplementary Figure 7. FimH binding pocket sequence is conserved in prostatitis isolates CP1, P10, and P16. The amino acid sequences of FimH from UPEC strains UTI89, NU14, 536, and CFT073, as well as from bacterial prostatitis isolates CP1, P10, and P16, were compared. No mutations were found in the binding pocket region (highlighted in blue). Common UPEC-specific SNPs are indicated in red.

Supplementary Figure 8

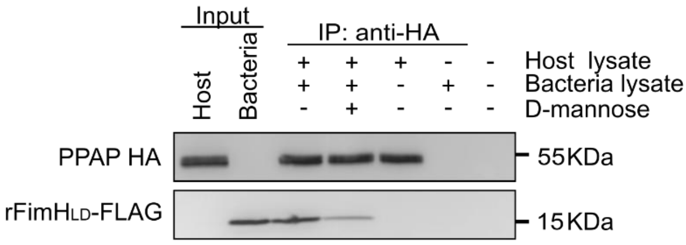

Supplementary Figure 8. rFimH$_{LD}$-FLAG is co-immunoprecipitated with rPPAP-HA *in vitro*. Representative image of co-IP of rFimH$_{LD}$-FLAG using the rPPAP-HA as bait (*n* = 3 biological replicates).

Supplementary Figure 9

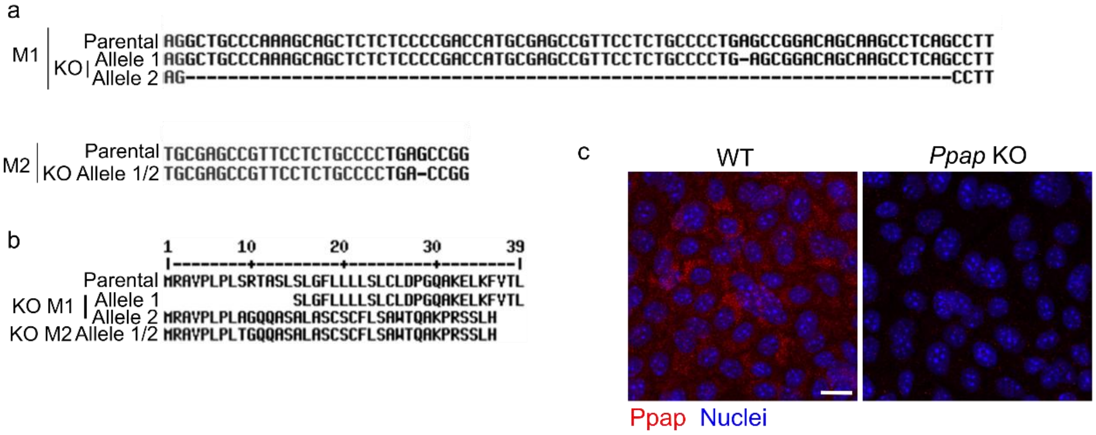

Supplementary Figure 9. Validation of *Ppap* CRISPR/Cas9 knockout organoid clones. **a**. Sanger sequencing shows a mutation in *Ppap* sequence for both KO mice (M denotes mouse). **b**. Amino acid sequence shows the sequence for the truncated proteins resulting from the KO. **c**. Representative images of HCR RNA-FISH analysis of *Ppap* on the organoid-based model (grown with DHT) for the WT parental and *Ppap* KO lines. Scale bar 25 µm (n = 1).

Source Data for Supplementary Figure 8

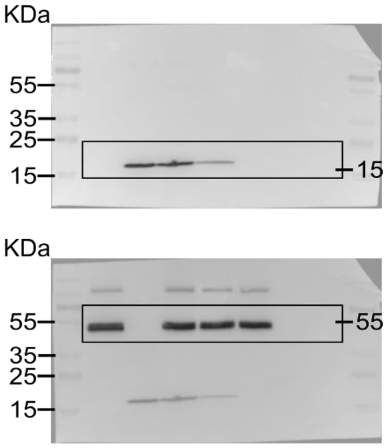

Source Data for Supplementary Figure 8. Uncropped blot.
