# Supplementary material for: Uropathogenic Escherichia coli invade luminal prostate cells via FimH–PPAP receptor binding
Source: Nat Microbiol. 2026 Jan 8;11(2):535–50. doi: 10.1038/s41564-025-02231-0 (PMC12872464; doi:10.1038/s41564-025-02231-0)
Supplement: Supplementary file 2 — Reporting Summary [file 41564_2025_2231_MOESM2_ESM.pdf]

nature portfolio

Corresponding author(s): Carmen Aguilar

Last updated by author(s): 12.11.2025

Reporting Summary

Nature Portfolio wishes to improve the reproducibility of the work that we publish. This form provides structure for consistency and transparency in reporting. For further information on Nature Portfolio policies, see our Editorial Policies and the Editorial Policy Checklist.

Statistics

For all statistical analyses, confirm that the following items are present in the figure legend, table legend, main text, or Methods section.

n/a	Confirmed	
☐	☒	The exact sample size (n) for each experimental group/condition, given as a discrete number and unit of measurement
☐	☒	A statement on whether measurements were taken from distinct samples or whether the same sample was measured repeatedly
☐	☒	The statistical test(s) used AND whether they are one- or two-sided<br>*Only common tests should be described solely by name; describe more complex techniques in the Methods section.*
☒	☐	A description of all covariates tested
☐	☒	A description of any assumptions or corrections, such as tests of normality and adjustment for multiple comparisons
☒	☐	A full description of the statistical parameters including central tendency (e.g. means) or other basic estimates (e.g. regression coefficient) AND variation (e.g. standard deviation) or associated estimates of uncertainty (e.g. confidence intervals)
☐	☒	For null hypothesis testing, the test statistic (e.g. F, t, r) with confidence intervals, effect sizes, degrees of freedom and P value noted<br>*Give P values as exact values whenever suitable.*
☒	☐	For Bayesian analysis, information on the choice of priors and Markov chain Monte Carlo settings
☒	☐	For hierarchical and complex designs, identification of the appropriate level for tests and full reporting of outcomes
☒	☐	Estimates of effect sizes (e.g. Cohen's d, Pearson's r), indicating how they were calculated

Our web collection on statistics for biologists contains articles on many of the points above.

Software and code

Policy information about availability of computer code

Data collection	Leica Application Suite Advance Fluorescence (LAS AF Leica Microsystems, v.2.7.3.9723), LAS X (v4.6.1.27508), ImageQuant LAS 4000, NovaSeq 6000 (Illumina), NovoCyte Quanteon flow cytometer.
Data analysis	Microsoft Excel (Microsoft Office, 2013), Prism (GraphPad, v.7.00), Inkscape (v1.3.2.),<br>ImageJ (v.1.50b), R (v4.3.0), Rstudio (v3.53), Phyton v3.8, LAS AF Lite (Leica, 2.6.0 build 7266), ImageQuant LAS 4000 Control Software, Cell Ranger (version v7.0.1.2), Seurat (v4.3.0), BioRender, NovoExpress software (Agilent).

For manuscripts utilizing custom algorithms or software that are central to the research but not yet described in published literature, software must be made available to editors and reviewers. We strongly encourage code deposition in a community repository (e.g. GitHub). See the Nature Portfolio guidelines for submitting code & software for further information.

Data

Policy information about availability of data

All manuscripts must include a data availability statement. This statement should provide the following information, where applicable:

- Accession codes, unique identifiers, or web links for publicly available datasets
- A description of any restrictions on data availability
- For clinical datasets or third party data, please ensure that the statement adheres to our policy

The data supporting the findings of this study have been deposited in Gene Expression Omnibus with the accession code GSE275482. All other relevant data are available from the corresponding author upon request.

Research involving human participants, their data, or biological material

Policy information about studies with human participants or human data. See also policy information about sex, gender (identity/presentation), and sexual orientation and race, ethnicity and racism.

Reporting on sex and gender	Only male patients tissue was used in this study. Ages 45-80.
Reporting on race, ethnicity, or other socially relevant groupings	Due to the low number of male patients used in this study, no discrimination based on age, race or genotypic information was applied. Samples from three donors were used (45-80 years old) were used. No financial compensation was provided.
Population characteristics	N/A
Recruitment	Patients that had planned a prostate surgery (e.g. biopsy for cancer screening or proctectomy) were informed by the doctor. Informed consent was obtained from all the donors.
Ethics oversight	Ethical committee of the University of Würzburg (Approval 168/22).

Note that full information on the approval of the study protocol must also be provided in the manuscript.

Field-specific reporting

Please select the one below that is the best fit for your research. If you are not sure, read the appropriate sections before making your selection.

☒ Life sciences ☐ Behavioural & social sciences ☐ Ecological, evolutionary & environmental sciences

For a reference copy of the document with all sections, see nature.com/documents/nr-reporting-summary-flat.pdf

Life sciences study design

All studies must disclose on these points even when the disclosure is negative.

Sample size	Mouse prostate organoids from 4 different mice (WT male C57BL/6 mice, 8-months-old). Experiments with human prostate tissue were done with samples from 3 donors.
Data exclusions	No data was excluded from the study
Replication	n for each experiment is stated in the figure legends.
Randomization	Human prostate tissue was chosen based on availability of tissue from patient at that time. Male mice for organoid generation were also randomly chosen from the pool of mice used at the moment for other experiments at the Institute.
Blinding	Blinding was not necessary in this study.

Reporting for specific materials, systems and methods

We require information from authors about some types of materials, experimental systems and methods used in many studies. Here, indicate whether each material, system or method listed is relevant to your study. If you are not sure if a list item applies to your research, read the appropriate section before selecting a response.

Materials & experimental systems

n/a	Involved in the study	
☐	☒	Antibodies
☐	☒	Eukaryotic cell lines
☒	☐	Palaeontology and archaeology
☐	☒	Animals and other organisms
☒	☐	Clinical data
☒	☐	Dual use research of concern
☒	☐	Plants

Methods

n/a	Involved in the study	
☒	☐	ChIP-seq
☐	☒	Flow cytometry
☒	☐	MRI-based neuroimaging

Antibodies

Antibodies used

All antibodies used in the study are commercially available and have been validated by the supplier.

Immunofluorescence:
Anti-Cytokeratin 5 (1:100, ab52635, Abcam)
Anti-Cytokeratin 5 Alexa Fluor® 647 antibody (1:100, ab193895, Abcam)
Anti-CD24a (1:100, 10600-1-AP, Proteintech)
Anti-ACPP (PPAP, 1:100, LS-C292593, LSBio)
Anti-KRT18 (1:100 ab133263, Abcam)
Anti-ZO-1 Polyclonal antibody (1:750, 21773-1-AP, Proteintech)
Anti-FLAG antibody (1:1,000; 66008-4-Ig, Proteintech)
Anti-E. coli LPS (1:500, ab35654, Abcam)
Flash Phalloidin™ Green 488 (1:250, 424201, Biolegend)
Flash Phalloidin™ Red 594 (1:250, 424203, Biolegend)
Phalloidin Alexa 647 (1:250, A30107, Invitrogen)
Hoechst 33342 (1:5,000, H3570, Life Technologies)
Anti-Rabbit IgG Alexa Fluor™ 488 (1:500, A21441, Invitrogen)
Anti-Mouse IgG Alexa Fluor™ 594 (1:500A21201, Invitrogen)

Western Blot:
Anti-mouse IgG-HRP conjugate (1:10,000, GENA931, Sigma Aldrich)
Anti-rabbit IgG-HRP conjugate (1:10,000, GENA934, Sigma Aldrich)
Anti-rabbit IgG-HRP conjugate (1:10,000, SA00001-2, Proteintech)
Anti-FLAG antibody (1:1,000; 66008-4-Ig, Proteintech)
Anti-HA Polyclonal Antibody (1:5,000, 51064-2-AP, Proteintech)
Anti-ACPP Antibody (1:300, HPA063916,Atlas Antibodies)
Anti-FimH Antibody (1:1,000, CSB-PA362349ZA01ENV, LSBio)
Anti-GroEL Antibody (1:5,000, G6532, Sigma)

Flow cytometry
Anti-mouse CD24 (FITC, 1:500, #101805, Biolegend)
Anti-mouse CD49f (Alexa647, 1:750, #313610, Biolegend)
Isotype controls Rat IgG2b, κ Isotype Ctrl (FITC, 1:500, #400633, Biolegend)
Isotype controls Rat IgG2a, κ Isotype Ctrl (Alexa 647, 1:750, #400526, Biolegend)

Bacterial Binding
Anti-HA (12.5 µg/mL, 51064-2-AP, Proteintech)
Anti-LPS (12.5 µg/mL, ab35654, Abcam)

Blocking assay
Anti-β1 integrin (1,5 µg/well, 6S6; sc-53711, Santa Cruz Biotechnology)
Anti-α3 integrin (1,5 µg/well, P1B5; sc-13545, Santa Cruz Biotechnology)

Validation

All antibodies used in this study were commercial antibodies available for Western-blot and immunofluorescence. Validation data are available on the manufacturer's websites and data sheets.

Eukaryotic cell lines

Policy information about cell lines and Sex and Gender in Research

Cell line source(s)	ATCC HTB-9 (5637), ATCC CCL-2.1 (HeLa229)
Authentication	Performed by ATCC
Mycoplasma contamination	Mycoplasma contamination was tested routinely every month.
Commonly misidentified lines (See ICLAC register)	None

Animals and other research organisms

Policy information about studies involving animals; ARRIVE guidelines recommended for reporting animal research, and Sex and Gender in Research

Laboratory animals	C57BL/6 mice WT, 8 months old, male.
Wild animals	N/A
Reporting on sex	Only male mice were used to generate organoids (females do not have prostates).
Field-collected samples	N/A
Ethics oversight	To adhere to the 3R principles (Replacement, Reduction, Refinement), we utilised only leftover material from mice previously used for other approved experiments.

Note that full information on the approval of the study protocol must also be provided in the manuscript.

Plants

Seed stocks	*Report on the source of all seed stocks or other plant material used. If applicable, state the seed stock centre and catalogue number. If plant specimens were collected from the field, describe the collection location, date and sampling procedures.*
Novel plant genotypes	*Describe the methods by which all novel plant genotypes were produced. This includes those generated by transgenic approaches, gene editing, chemical/radiation-based mutagenesis and hybridization. For transgenic lines, describe the transformation method, the number of independent lines analyzed and the generation upon which experiments were performed. For gene-edited lines, describe the editor used, the endogenous sequence targeted for editing, the targeting guide RNA sequence (if applicable) and how the editor was applied.*
Authentication	*Describe any authentication procedures for each seed stock used or novel genotype generated. Describe any experiments used to assess the effect of a mutation and, where applicable, how potential secondary effects (e.g. second site T-DNA insertions, mosiacism, off-target gene editing) were examined.*

Flow Cytometry

Plots

Confirm that:

☒ The axis labels state the marker and fluorochrome used (e.g. CD4-FITC).

☒ The axis scales are clearly visible. Include numbers along axes only for bottom left plot of group (a 'group' is an analysis of identical markers).

☒ All plots are contour plots with outliers or pseudocolor plots.

☒ A numerical value for number of cells or percentage (with statistics) is provided.

Methodology

Sample preparation	For analysis of naïve cells, 7days-old 2D models were dissociated into single cells with TrypLE Express (12605028, Gibco) and washed with FACS buffer (PBS, 10% FBS, 50mM EDTA). Infected cells were treated washed 3 times with PBS and then stained following the same protocol.
Instrument	NovoCyte Quanteon flow cytometer.
Software	NovoExpress software (Agilent).
Cell population abundance	No sorting was performed. The total population after cell debris exclusion was separated into Cd24a+ or Cd49+ cells. Both populations were well represented in all the samples.
Gating strategy	Cells were first gated on SSC versus FSC to exclude debris, then gated based on APC-Cd49f (basal cells) or FITC-Cd24 (luminal cells) expression. mCherry-positive cells (infected cells) were subsequently measured. Gating strategy is shown in Supplementary Figure 5.

☒ Tick this box to confirm that a figure exemplifying the gating strategy is provided in the Supplementary Information.
