# Supplementary figures and images for: Uropathogenic Escherichia coli invade luminal prostate cells via FimH–PPAP receptor binding
Source: Nat Microbiol. 2026 Jan 8;11(2):535–50. doi: 10.1038/s41564-025-02231-0 (PMC12872464; doi:10.1038/s41564-025-02231-0)

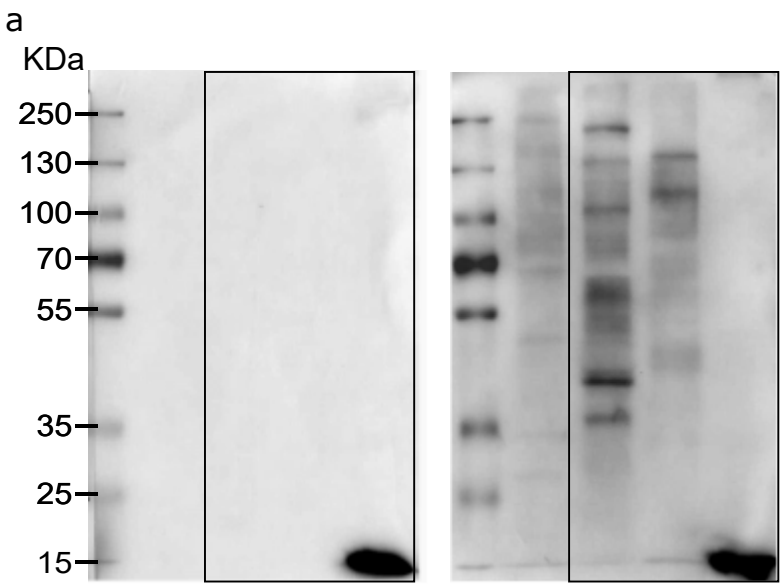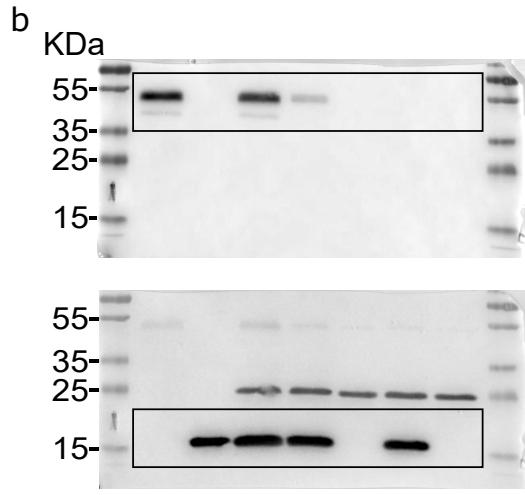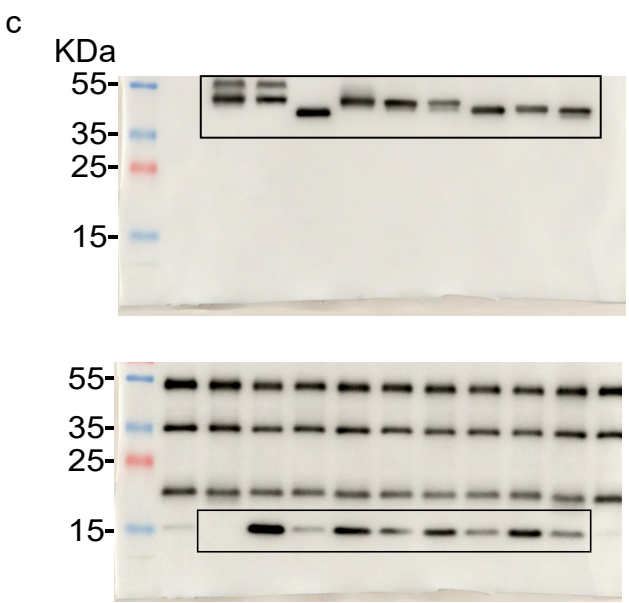

Supplement: Supplementary file 5 — Uncropped western blots corresponding for Fig. 4d–f. [file 41564_2025_2231_MOESM5_ESM.pdf]

KDa

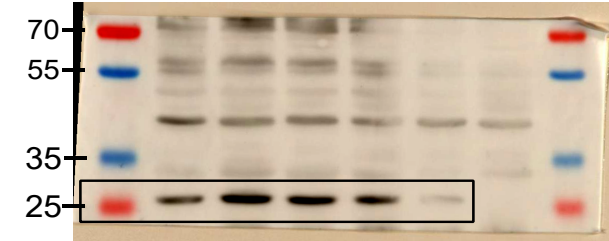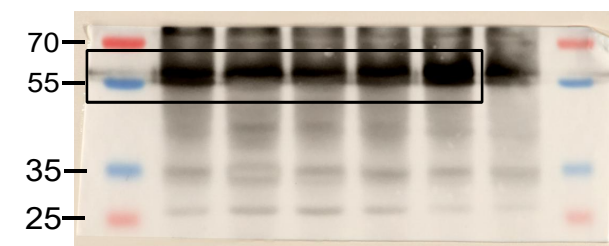

Supplement: Supplementary file 6 — Uncropped western blot for Extended Data Fig. 6d. [file 41564_2025_2231_MOESM6_ESM.pdf]
